# Enhancing the Production of the Phenolic Extracts of Asparagus Using an Advanced Green Process

**DOI:** 10.3390/metabo12100951

**Published:** 2022-10-06

**Authors:** Lucía López-Salas, Isabel Borrás-Linares, Rosa Quirantes-Piné, Tatiana Emanuelli, Antonio Segura-Carretero, Jesús Lozano-Sánchez

**Affiliations:** 1 Department of Food Science and Nutrition, University of Granada, Campus Universitario s/n, 18071 Granada, Spain; 2 Department of Analytical Chemistry, Faculty of Sciences, Avda Fuentenueva s/n, University of Granada, 18071 Granada, Spain; 3 Research and Development Functional Food Centre (CIDAF), Health Science Technological Park, Avenida del Conocimiento 37, EdificioBioRegión, 18016 Granada, Spain; 4 Department of Food Technology and Science, Center of Rural Sciences, Federal University of Santa Maria, Camobi, Santa Maria 97105-900, Brazil

**Keywords:** asparagus, extraction, phenolic compounds, optimization, pressurized liquid extraction, HPLC–ESI-TOF-MS

## Abstract

*Asparagus officinalis* L. is a common vegetable widely consumed due to its high consumer acceptance. In addition to its flavor, green asparagus contains a high amount of bioactive compounds with health-promoting effects. In this sense, the growing concern of the public health system to promote a diet with a higher consumption of vegetables makes research on phytochemicals from this food of interest. In order to study the content of bioactive compounds from plant matrices, the combination of advanced extraction and analytical techniques within the context of green chemistry is an indispensable working model in today’s research. In the present experimental work, the composition of the phytochemicals of green asparagus from the Protected Geographical Indication (PGI) located in Huétor Tájar, Granada (Spain), was evaluated by environmentally friendly extraction techniques. In order to carry out this work, the recovery of bioactive compounds was evaluated by pressurized liquid extraction (PLE) using GRAS (Generally Recognized As Safe) solvents (mixtures of water and ethanol). The extraction was optimized using a Response Surface Methodology (RSM) based on a 2^4^ factorial Central Composite Design (CCD). The experimental model was followed by high-performance liquid chromatography coupled to electrospray ionization time-of-flight mass spectrometry (HPLC–ESI-TOF-MS) analytical methodology for a comprehensive characterization. The optimized methodology was compared with conventional solid–liquid extraction protocols using ethanol and water. The results highlighted the potential of advanced PLE techniques compared to conventional systems for the recovery of green asparagus phytochemicals. Moreover, the analytical characterization allowed the identification and quantitation of major phenolic compounds belonging to phenolic acids and flavonoids families. Therefore, an easy, fast, and novel methodology to optimize the extraction of bioactive compounds from green asparagus has been optimized, using Green and GRAS methodology, which enables a better understanding of the bioactive composition of this widely consumed food.

## 1. Introduction

Epidemiological studies show a relationship between the consumption of vegetables and the reduction of chronic diseases, pointing out that phytochemicals present in vegetables are the components responsible for this protective effect [1]. These bioactive properties have been partly related to high antioxidant activity, and the main compounds responsible for this function are polyphenols [2,3]. Therefore, in the current society, where pathologies associated with an unhealthy diet are becoming a global concern, the study of the presence of bioactive compounds in foods, and concretely their phenolic composition, is interesting due to their beneficial effects.

For this purpose, in recent years the scientific community has developed novel research lines within a green chemical model regarding extraction methodologies in order to obtain bioactive-enriched products of interest from different organic matrices, i.e., supercritical fluids, microwave-assisted, ultrasound-assisted or pressurized fluid extraction [4,5,6,7]. Among these advanced technologies, extraction using pressurized fluids (PLE) is a very useful technique in the case of food samples, facilitating and improving the extraction of polar compounds from plant matrices. The most important advantages of this novel technique include high yields, reduced extraction time, low solvent consumption, and the use of cheap and environmentally friendly solvents, such as water and ethanol. All of these advantages explain why many authors have applied PLE as the extraction technique in their research. Woloszyn et al., applied PLE to obtain antioxidant extracts from *Mentha pulegium*, *Equisetum giganteum,* and *Sida cordifolia*, for evaluating the yield, total phenolic content (TPC), and flavonoid content [8]. In the same way, Ho et al., optimized the PLE methodology for TPC, gallic acid, and p-hydroxybenzoic acid (PHBA) from *Pseuderanthemum palatiferum* (Nees) Radlk. leaves using response surface methodology [9]. Furthermore, Dobroslavić et al. conducted research to determine optimal conditions for the PLE of laurel leaf polyphenols, and the extract obtained under optimal PLE conditions showed flavonols as the most abundant compounds [10]. Katsinas et al. carried out PLE of supercritical defatted olive pomace [11]. In this study, the authors were able to increase the extraction of phenolic compounds such as decarboxymethyl oleuropein aglycone, hydroxytyrosol, and tyrosol compared to conventional extraction methods. Moreover, Barrales et al. recovered phenolic compounds from citrus by-products using pressurized liquids, and the results showed nine flavonoids, with three that were glycosylated and two hydroxybenzoic acids [12]. Those previous results pointed out the potential of PLE to recover phenolic compounds from vegetable food matrices and supports its choice for the present study.

The *Asparagus officinalis* L. variety from the Huétor Tajar region (Granada, Spain) was registered under Protected Geographical Indication (PGI) through the European Union Regulation [13]. This asparagus were obtained from native varieties selected in the area since the beginning of the last century. This vegetable belongs to the Asparagaceae family, being commonly consumed around the world due to its high nutritional value and high mineral content, vitamin, amino acid, and dietary fiber contents [14]. In addition to these well-known nutritional properties, it can also help to prevent numerous pathologies such as cancer, cardiovascular or inflammatory diseases [15]. Concretely, asparagus sprouts are known to contain large amounts of rutin, a flavonoid with remarkable anti-inflammatory, anti-tumor, antibacterial, and antiviral activities [16,17]. Therefore, a better understanding of the bioactive composition and peculiarities of this PGI asparagus variety, which is exported and consumed in numerous countries, especially those of the European Union, including northern countries, will help to promote its consumption among this population due to its potentially beneficial bioactive content.

In this scenario, PLE green technology could provide a new standpoint to study the bioactive composition of this widely consumed food from the Huétor Tajar region. Therefore, the present work was focused on the study of the phenolic compounds in green asparagus, optimizing the PLE methodology and comparing it with conventional solid–liquid extraction. The obtained extracts were characterized by high-performance liquid chromatography coupled to electrospray ionization time-of-flight mass spectrometry (ES) for a comprehensive characterization of its bioactive chemical profile.

## 2. Materials and Methods

### 2.1. Plant Material and Sample Treatment

#### 2.1.1. Plant Material

Plant material used in this study consisted of samples of green asparagus from the Andalusian cooperative Centro Sur S.C.A (Huétor-Tájar, Granada, Spain). Samples of the *Asparagus officinalis* L. variety were collected in three geographical regions within the Huétor-Tájar PGI area (Huétor Tájar, Íllora, and Loja locations) during the collection campaign (April 2021). To carry out the experiments, a homogeneous sample of the three geographical areas was obtained and refrigerated until processing.

#### 2.1.2. Freeze-Drying and Grinding

Prior to the lyophilization process, asparagus samples were cut into pieces of an approximate size of 2 cm. Then, they were lyophilized using a SP Scientific Virtis AdVantage 2.0 BenchTop Freeze Dryer lyophilizer, with an ice condensation capacity of 4 L in 24-h cycles. The total freeze-drying process was 92.5 h.

Once the samples were lyophilized, they were subjected to grinding to reduce the particle size and increase the contact surface with the solvent, improving the efficiency of the extraction process. Simultaneous grinding and sieving of the samples were carried out in a ZM 200 ultracentrifugal mill (Retsch, Spain). The grinding was carried out using a 1 mm sieve to obtain a sample with an optimal particle size for the selected extraction technique, as 80% of the total ground sample reached a size less than half of the mesh opening of the used annular sieve. The ground and sieved samples were stored at room temperature and were protected from light and humidity until the extraction procedure. 

### 2.2. Reagents

All reagents used during the development of this work were of analytical reagent grade. For extraction processes, water used as a solvent was purified by a Milli-Q system from Millipore (Bedford, MA, USA), and ethanol was purchased from VWR Chemicals (Radnor, PA, USA). Sand and cellulose filters were obtained from Fisher Chemicals (Waltham, MA, USA). Acetic acid and acetonitrile for the HPLC analysis were purchased from Sigma–Aldrich (Steinheim, Germany) and Fisher Chemicals (Waltham, MA, USA), respectively. The standards for the calibration curves (chlorogenic acid, quinic acid, ferulic acid, quercetin-3-rutinoside, and quercetin-3-glucoside) were acquired from Sigma Aldrich (Steinheim, Germany).

### 2.3. Phenolic Compound Extraction

The extraction of phenolic compounds was carried out using two different methodologies: (a) Conventional Solid–liquid extraction (SLE); and (b) Pressurized fluid extraction (PLE).

#### 2.3.1. Conventional Solid–Liquid Extraction (SLE)

In order to carry out the SLE extraction, 1 g of lyophilized sample was mixed with 15 mL of the solvent mixture (ethanol:water, 70:30, *v*/*v*). Samples were shaken in darkness in an orbital shaker for a total time of 30 min at a shaking speed of 350 min^−1^. After that, the samples were sonicated for 15 min under refrigerated conditions at atmospheric pressure and then centrifuged at 12,500 rpm for 15 min at 4 °C. The supernatants were collected and filtered through 0.2 µm regenerated cellulose filters. Solvents were evaporated at room temperature under vacuum on a SpeedVac Savant vacuum concentrator (Thermo Scientific, Waltham, MA, USA). The extraction process was carried out in triplicate to ensure the reproducibility of the process.

#### 2.3.2. Pressurized Liquid Extraction (PLE)

The optimization of the extraction process using pressurized fluids was carried out through a response surface experimental design using the Statgraphics Centurion XV software, version 15.1.02. The applied model was a 2^4^ factorial composite central design. The four factors established as independent variables were the extraction temperature (in a range of 60–160 °C), the extraction time (10–30 min), percentage of ethanol in the extraction solvent (20–80%), and the sand–sample ratio (2–5, *w*/*w*). For each variable, two levels were considered, maximum and minimum. Additional experiments, called star or axial points, were located below and above these maximum and minimum values, allowing the determination of the response surface curvature for each of the factors. The generated experimental design consisted of a total of 25 experiments that were performed in a randomized order (Table S1). The response variables were the extraction yield and the phenolic composition of the extracts determined by HPLC–ESI-TOF-MS. 

The equipment used for PLE was an ASE ™ 350 from Dionex (Sunnyvale, CA, USA). Solvents were sonicated prior to extraction to remove dissolved air to prevent any possible degradation of compounds through oxidation reactions. In all experiments, the extraction was performed in static mode at 1500 psi, with a variable extraction time and temperature indicated in Table S1. To carry out the extraction process, a 34 mL extraction cell was prepared using a sandwich configuration: 15× *g* of sand + 1 g of sample plus the corresponding amount of sand according to the sand:sample ratio (*w*/*w*) of each experimental point (Table S1) +20 g of sand on the top of the cell. Cellulose filters were placed in both the upper and lower part of the cell to prevent possible blockage of the system. Once the extracts were obtained, the same procedure was applied as described in the previous section for conventional solid–liquid extracts consisting of centrifugation and evaporation steps.

### 2.4. HPLC–ESI-TOF-MS Analysis

The analyses of the SLE and PLE extracts were carried out with a RRLC 1200 series liquid chromatograph (Agilent Technologies, Palo Alto, CA, USA), equipped with a vacuum degasser, a binary pump, an autosampler, a thermostatted column compartment, and a diode array detector (DAD).

The analytical column used for separation was a Zorbax Eclipse Plus C18 1.8 μm, 150 × 4.6 mm, (Agilent Technologies, Palo Alto, CA, USA). The used mobile phase was water with 0.5% acetic acid as eluent A and acetonitrile as eluent B. The flow rate was set at 0.5 mL min^−1^, the injection volume was 10 µL, and the column temperature was kept at 25 °C. The total analysis run was 50 min, using the following multi-step linear gradient: 0 min, 5% B; 40 min 100% B; 45 min 5% B; and finally, a conditioning cycle of 5 min with the initial conditions (5% B) before the next injection to equilibrate the system.

The HPLC system was coupled to a Bruker Daltonik GmbH (Bremen, Germany) microTOF time-of-flight mass spectrometer equipped with an ESI interface (G1607 model from Agilent Technologies, Palo Alto, CA, USA). To achieve a stable spray at the interface, the effluent from the HPLC column was reduced using a “T” type splitter before entering the mass spectrometer (1:3 division ratio). Thus, the flow that reached the TOF-MS detector was 125 μL min^−1^.

The analyses were carried out under negative polarity considering a mass range of 50–1000 *m*/*z*. The values of the source parameters were as follows: capillary voltage of +4.5 kV; drying gas temperature, 190 °C; drying gas flow, 9 L min^−1^; and nebulizer gas pressure, 2.0 bar. The values for the transfer parameters were as follows: capillary output voltage, −150 V; skimmer voltage 1, −50 V; hexapole voltage 1, −23 V; hexapole radio frequency, 100 Vpp; and skimmer 2 voltage, −22.5 V.

The instrument was externally calibrated with a Cole Palmer 74,900–00-05 syringe pump (Vernon Hills, IL, USA) that was connected directly to the interface and contained a 10 mM sodium acetate solution. The mixture was injected at the beginning of each analysis, and all spectra were calibrated prior to the identification of compounds. The exact mass data of the molecular ions were processed through DataAnalysis 4.0 software (Bruker Daltonics, Bremen, Germany), which provided a list of possible elemental formulas using the Generate-Molecular Formula Editor.

To carry out the identification and quantification of the detected analytes in all of the asparagus extracts (SLE and PLE), the samples were prepared at a concentration of 10 mg/L using the corresponding hydro-alcoholic mixture used for their extraction as a solvent. Triplicate injections were made of each extraction condition. To carry out a quantitative analysis, calibration curves were prepared from seven commercially available standards, some of them present in the extracts and others with similar structures to other identified compounds: chlorogenic acid, quinic acid, ferulic acid, quercetin-3-rutinoside, and quercetin-3-glucoside.

Stock solutions of each of these standards were prepared at a concentration of 1000 mg/L. Table S2 summarizes the calibration curves and parameters. All standards showed good linearity between the different concentration ranges. The concentration of phenolic compounds was calculated using the individual area in the chromatogram of each compound with interpolation using the corresponding calibration curve of the commercial standard.

## 3. Results

### 3.1. Characterization and Quantification of Phenolic Compounds in PLE and SLE Extracts by HPLC–ESI-TOF-MS

**Figure 1:**
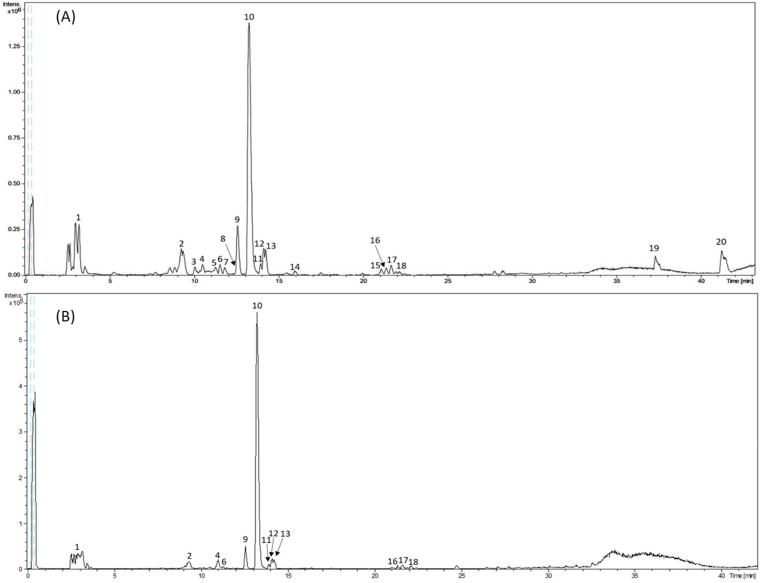
Representative base peak chromatogram of the HPLC–ESI-TOF-MS analysis of phenolic extracts from asparagus: (**A**) PLE 6, (**B**) SLE.

Representative base peak chromatograms of the analyzed extracts are shown in Figure 1. The detected peaks are numbered according to their retention time.

Once the analysis of the samples was carried out, the detected polar compounds were tentatively identified by comparing their retention time and mass spectra with the available commercial standards or by means of the information reported in databases and bibliography. A total of 20 compounds were identified in the samples, with the majority being phenolic acids and flavonoids, whereas four of them remain unknown despite efforts for their identification (UK1-4).

The observed peaks in the chromatogram of Figure 1 correspond to those putatively identified in Table 1, in which the tentatively identified compounds are proposed with their retention time, experimental and theoretical *m*/*z*, error, mSigma value, and molecular formula.

As can be observed in Figure 1, the intensity of the peaks in the SLE extract (intens. ×10^5^) was much lower than that in the PLE (intens. ×10^6^). Likewise, it can be seen that many of the compounds detected in the PLE could not be detected in the SLE sample, highlighting the advantages of pressurized fluid extraction compared to conventional extraction in order to recover polyphenols. The HPLC–ESI-TOF-MS analysis of the SLE extracts pointed out that all compounds detected in the SLE extraction were also identified in the PLE extracts.

Most of the detected compounds have been previously described in asparagus [3,18,19,20,21]. With regard to phenolic acids, seven compounds were characterized. Peak 2, with molecular formula C_16_H_18_O_9_, was identified as chlorogenic acid. Peak 3, with a retention time of 10.5 min, was proposed as coumaroylquinic acid. This compound was not able to be extracted by all PLE conditions, being not detected in PLE 20 (160 °C, 20% ethanol, 30 min and 2 S–S) with SLE extraction. Peak 4 yielded a deprotonated molecule at *m*/*z* 367 and was characterized as feruloylquinic acid. Peak 6, with a retention time of 11.5 min and with *m*/*z* 355, was identified as feruloyl hexose. This peak was detected under all extraction conditions except for PLE 20 (160 °C, 20% ethanol, 30 min and 2 S–S), PLE 21 (160 °C, 20% ethanol, 10 min and 2 S–S), and PLE 22 (160 °C, 20% ethanol, 10 min and 5 S–S). Peak 16, with *m*/*z* 383 and molecular formula C_21_H_20_O_7_, was described as dicoumaroylglycerol; whereas peak 17, with a retention time of 21.6 min, was characterized as coumaroylferuloyl glycerol. Finally, peak 18, with a retention time of 21.9 min and *m*/*z* 443 was proposed to be diferuloyl glycerol.

Furthermore, five compounds were tentatively identified as flavonoids. Peak 9, with *m*/*z* 771, was characterized as quercetin glucosyl rutinoside. Peak 10, eluted at 13.2 min and *m*/*z* 609, putatively corresponded to rutin. Peak 11, with molecular formula C_21_H_20_O_12_, was proposed as quercetin glucoside; while peak 12, eluting at 14.1 min and with the molecular formula C_27_H_30_O_15_, was identified as kaempferol rutinoside. Peak 13, with a retention time of 14.2 min, corresponded to isorhamnetin rutinoside.

Other non-phenolic compounds identified in extracts were peaks 1, 15, 19, and 20. Peak 1, with a retention time of 3.00 min and *m*/*z* 191, was identified as quinic acid. Furthermore, peak 15 was identified as trihydroxy octadecaenoic acid (oxylipin) and finally, peaks 19 and 20 were identified as linolenic and linoleic acids (fatty acids), respectively.

**Figure 2:**
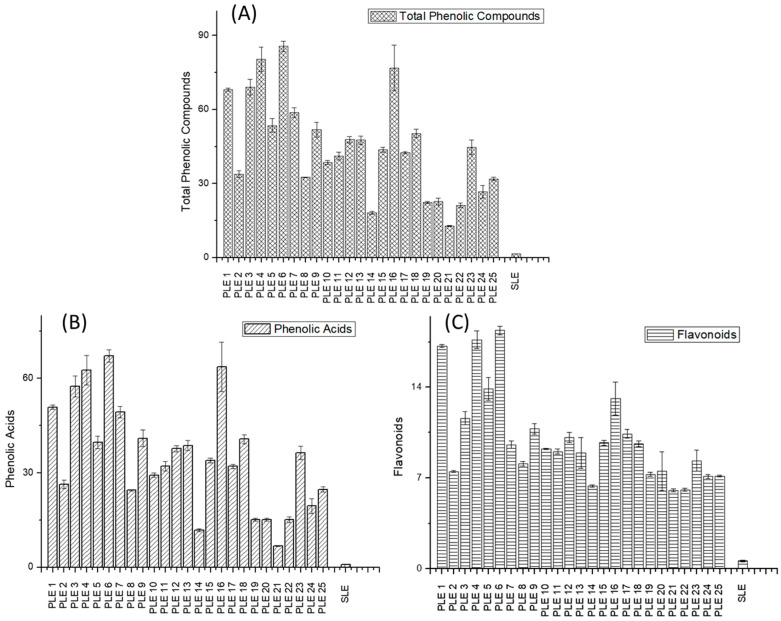
Total content of phenolic compounds (**A**), phenolic acids (**B**), and flavonoids (**C**) of the PLE and SLE extracts. Values expressed in mg compounds/g extract as X ± SD.

Regarding the quantification of these phytochemicals, Figure 2 depicted the total phenolic, total phenolic acid, and total flavonoid contents under each PLE and SLE conditions. The concentrations were calculated as the sum of the individual phenolic compounds (Table S4) and expressed in mg of compound per gram of extract as the mean ± standard deviation value (X ± SD). Differences between the two extraction techniques in terms of the recovery capacity of phenolic compounds were very marked, as the SLE extraction recovered fewer compounds in all cases. For example, feruloylquinic acid, feruloyl hexose, and rutin were the most abundant compounds in all PLE extracts. However, the extraction of these compounds by conventional extraction was very low (up to 10 times lower). A similar behavior could be observed in the other compounds when comparing the two extraction techniques. Coumaroylquinic acid was not detected in SLE extracts while it was extracted under practically all PLE conditions.

In regards to the total phenolic compounds of the *Asparagus officinalis* L. PLE extracts, the PLE 6 (60 °C, 80% ethanol, 10 min and 5 S–S), PLE 4 (60 °C, 80% ethanol, 30 min and 5 S–S), and PLE 16 (110 °C, 94.48% ethanol, 20 min and 3.50 S–S) conditions showed the highest concentration. According to the present results, a high percentage of ethanol in aqueous mixtures, both long and short extraction times, as well as a moderate to high extraction temperature and sand–sample ratio, enhanced the extraction of these compounds. In general, extraction conditions with the most extreme temperatures above 160 °C (PLE 18–25) were able to recover a lower content of phenolic compounds, except for the PLE 18 and PLE 23 conditions. This decrease in the concentration of phenolic compounds may be due to the degradation of these types of compounds as a consequence of the high extraction temperatures.

The analysis of the total phenolic acid composition indicated that the highest recovery was obtained under the conditions PLE 6 (60 °C, 80% ethanol, 10 min and 5 S–S), PLE 16 (110 °C, 94.48% ethanol, 20 min and 3.50 S–S), and PLE 4 (60 °C, 80% ethanol, 30 min and 5 S–S). These conditions are in agreement with those that provided the highest concentration of total phenolic compounds, as indicated above.

On the other hand, the analysis of the total flavonoid content showed that PLE 6 (60 °C, 80% ethanol, 10 min and 5 S–S), PLE 4 (60 °C, 80% ethanol, 30 min and 5 S–S), and PLE 1 (35.87 °C, 50% ethanol, 20 min and 3.50 S–S) were the best conditions for this family of compounds. The best two conditions considering the extraction of flavonoids were the same as those for the total phenolic compounds. However, the third best condition with the highest extraction of these compounds was PLE 1, which is not considered among the top three for total phenolic compounds or phenolic acids.

From the quantitative composition point of view, as mentioned before, it is important to highlight that when comparing this extraction methodology with the values reported in Table S4 for the PLE extracts, all of the phenolic compounds were recovered in a lower concentration. The content of total phenolic compounds of the SLE was 1.52 ± 0.04 mg compounds/g extract, a much lower value than that obtained for all PLE extracts. The same phenomenon could be observed for the values obtained for phenolic acid (0.91 ± 0.02 mg compounds/g extract), and flavonoid (0.61 ± 0.06 mg compounds/g extract) families (Table S4). Those results also pointed out the potential for the recovery of bioactive compounds from green asparagus compared to conventional procedures.

### 3.2. PLE Optimization of Green Asparagus

PLE methodology was optimized following the procedure described in Section 2.3.2. Table 2 summarizes the statistical analysis of the proposed model including linear, quadratic, and interactions effects of independent variables (X_1_: Temperature, X_2_: %EtOH, X_3_: Extraction time and X_4_: S–S, sand–sample ratio) on the variable responses: extraction yield (Y_1_), total phenolic compounds (Y_2_), phenolic acids (Y_3_), and flavonoids (Y_4_).

The extraction yield was calculated as the weight of collected asparagus dry extract per lyophilized asparagus material used in the extraction procedure (*w*/*w*, in grams).

**Figure 3:**
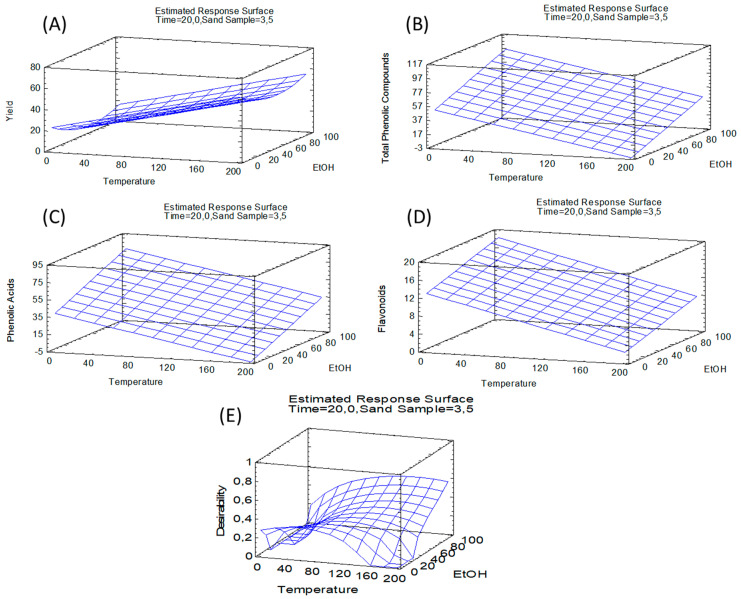
Surface response graphs: (**A**) extraction yield; (**B**) total phenolic compounds; (**C**) phenolic acids; (**D**) flavonoids, and (**E**) optimization of multiple responses (yield and total phenolic compounds).

The lack-of-fit test verified the fitting quality of the applied model, showing that the model was fitted for all response variables, as the value of this test was not significant (*p*-value higher than 0.05). The R^2^ values for each variable indicated that the adjusted model explained 71.9%, 91.9%, 90.9%, and 89.1% of the variability in yield, total polyphenols, total phenolic acids, and total flavonoids, respectively. As the model was satisfactory, it was used to generate the regression equations and for the creation of surface response graphs (Figure 3).

Significant effects were showed by those independent variables or interactions, which had a *p*-value equal to or less than 0.05. Therefore, for yield, the linear variables with a significant effect were extraction temperature and time, while the significant quadratic variables were ethanol–ethanol, the ethanol–sand sample, the time–sand sample, and the sand sample–sand sample.

Taking into consideration the phenolic composition, for all chemical families, temperature and ethanol percentage were significant. With regard to the other independent variables, the analysis of the results pointed out that only the quadratic effect of the ethanol–sand sample ratio had statistical significance for flavonoid recovery. The fitted equations of the model for each response variable are described by Equations (1)–(4).

(1) $$\begin{array}{cc} Y1=89.979\;+ & 0.195133\;X_{1\;}-0.923551\;X_{2\;}-0.684708\;X_{3\;}-28.849\;X_{4\;}\\ & +\;0.00430331\;X_{2}^{\;\;2}+0.126381\;X_{2}X_{4}+0.305976\;X_{3}X_{4}\\ & +\;2.23455\;X_{4}^{\;\;2} \end{array}$$

(2) $$Y2=47.0122-0.267797\;X_{1\;}+0.461685\;X_{2\;}+0.0848633\;\;X_{3\;}+0.737174\;X_{4\;}$$

(3) $$Y3=34.9672-0.217163\;X_{1\;}+0.402736\;X_{2\;}+0.103295\;\;X_{3\;}+0.471984\;X_{4\;}$$

(4) $$\begin{array}{cc} \;Y4=18.6407 & -\;0.0529933\;X_{1\;}-0.0710436\;X_{2\;}-0.028264\;\;X_{3\;}-1.46456\;X_{4\;}\\ & +\;0.0361349\;\;X_{2}X_{4} \end{array}$$

**Equations of the fitted model**: (a) extraction yield (Y1); (b) content of total phenolic compounds (Y2); (c) content of phenolic acids (Y3); and (d) content of flavonoids (Y4).

By replacing the values of the independent variables in the equation, it was possible to estimate the theoretical value for each response variable. Table 3 shows the comparison between experimental results and predicted values for PLE. A low value (<10%) of the coefficient of variance (CV) indicates a good reproducibility of the investigated systems, whereas CV values between 11 and 20% indicates an acceptable variation [22]. Therefore, most of the obtained CV values showed good or acceptable reproducibility, and only a few of them exceeded the 20% threshold.

The optimization of extraction conditions to maximize the response variables was evaluated. Table 4 shows the theoretical values of the independent variables that maximized the response variables provided by the model. The optimum conditions of PLE yield from *Asparagus officinalis* L. were found at 184 °C, 94.5% ethanol, 35 min and a sand–sample ratio of 5.7, with a yield of 89.5%.

In all cases, optimum PLE conditions for recovering phenolic compounds were about 36 °C, between 94.1 and 94.5% ethanol, and a sand–sample ratio between 5.2 and 5.7. Only in the case of the extraction time could a clear difference be observed with flavonoids (5.8 min) by comparing this variable with both phenolic acids and total phenolic compounds (approximately 30 min). The optimum temperature for phenolic compound extraction was much lower than the one proposed to optimize the extraction yield, which can be explained by the thermolabile behavior of phenolic compounds and the possible co-extraction of other kind of compounds at higher temperatures that increase the extraction yield [23]. The optimum values for the rest of the variables (% ethanol, time, and sand–sample ratio) were similar for both response variables, extraction yield, and the recovery of phenolic compounds.

Finally, the theoretical values that jointly maximized the response variables of yield and total phenolic compounds were 67.7 °C, 93% ethanol, 34.8 min, and a sand–sample ratio of 5.7. The optimum theoretical yield and total phenolic compounds were 66.4% and 78.94 mg/g, respectively. These values of the independent variables were more or less similar to those applied under the PLE 4 condition (60 °C, 80% ethanol, 30 min, and 5 S–S), which resulted in a yield of 50.25% and 80 mg/g of phenolic compounds.

By comparing the multiple response values with individual optimum values for yield and total phenolic compounds, it was found that temperature was the most critical parameter. To achieve a balance and maximize both responses, it would be necessary to apply an intermediate temperature between the optimum of each response variable. Therefore, a high yield would be obtained without destroying thermolabile compounds, such as the target compounds. The rest of the multiple response values (% ethanol, time, and S–S ratio) were very similar to the optimum value for both yield and total phenolic compounds.

Despite that the well-known main crucial factor that affects the extraction of phenolic compounds is the matrix that contains them, it should be highlighted that the present results for the PLE optimization of total phenolic compound recovery were in agreement with those obtained for other vegetable matrices. Indeed, the optimum temperature and ethanol percentage for the recovery of phenolic compounds from buriti (*Mauritia flexuosa* L.) shell were 71.21 °C and 92.43% of ethanol in aqueous mixture [24]. Although the optimum temperature for phenolic compound extraction from this matrix was double that compared to asparagus, both were within the range considered to be low extraction temperatures (below 100 °C). Furthermore, 71.21 °C is similar to 67.6 °C, which was proposed to maximize the multiple responses in asparagus samples. Concerning ethanol percentages, in both cases, the value was very high. It is important to note that the experimental temperature and ethanol percentages evaluated by these authors ranged from 28.79 °C to 71.21 °C and from 7.57% to 92.43%, respectively. However, neither the extraction time nor the sample–sand ratios were taken into account. In addition, another study on the PLE of phenolic compounds in *Passiflora* leaves showed similar results, and the optimum extraction conditions were 80 °C and 64% ethanol [25]. The temperatures and hydroalcoholic mixtures considered in this study were 40, 60, and 80 °C and 40, 70, and 50% ethanol. The obtained results were also compared to other studies that used different extraction conditions with no optimization step. For example, the recovery of phenolic compounds from citrus peel using PLE was evaluated through absolute ethanol and hydroalcoholic mixtures (75% and 50% ethanol) with PLE solvent at temperatures of 45, 55, and 65 °C [12]. The authors concluded that the best extraction conditions were 65 °C and 75%.

**Figure 4:**
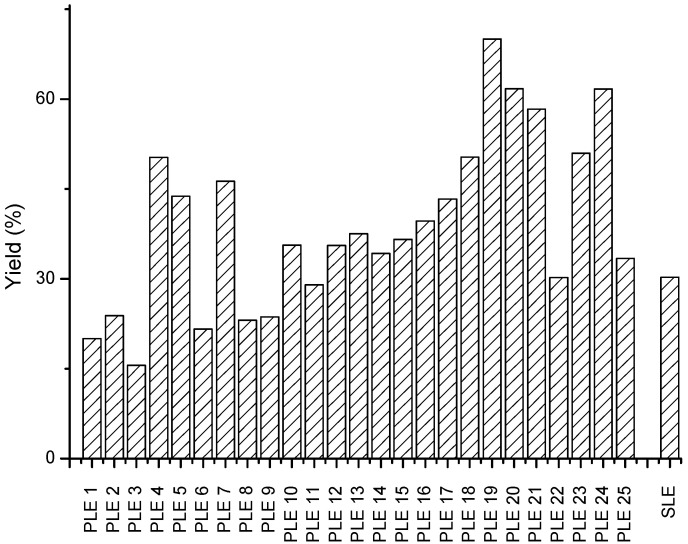
Extraction yield of different PLE experiments (1–25) and SLE extraction.

On the other hand, the obtained results for the PLE of green asparagus were compared to those obtained for conventional extraction. Figure 4 and Table S3 show the extraction yield obtained by PLE and SLE. This variable reached an equal or higher value than 50% for PLE 19 (160 °C, 20% ethanol, 30 min, and 5 S–S), PLE 20 (160 °C, 20% ethanol, 30 min, and 2 S–S), PLE 21 (160 °C, 20% ethanol, 10 min, and 2 S–S), and PLE 24 (160 °C, 80% ethanol, 10 min, and 2 S–S). Among these, condition PLE 19 was the one that showed the highest yield (70.01%). The extraction yield for the SLE condition was 30% (Figure 4, Table S3). This value was two times lower than the best condition of PLE 19.

Under the same behavior, the obtained values pointed out that these results were in agreement with those described for the PLE technique in the case of other vegetables matrices. For example, extraction yields were similar to those obtained for the PLE of *Lippia citriodora* leaves and passion fruit rinds [26,27]. According to the obtained results, a higher extraction temperature enhances the extraction of these compounds. This fact could be explained by the increase of solvent diffusivity with increasing temperature, enhancing the extraction of several compounds from plant matrices [28]. However, it was not possible to establish a relationship between the percentage of ethanol, extraction time, and S–S with the extraction yield. These results highlighted the potential of PLE for the recovery of green asparagus phytochemicals, which is in concordance with the results reported by other authors.

## 4. Conclusions

In view of the obtained results, the potential of PLE as a procedure to obtain *Asparagus officinalis* L. phenolic compound-enriched extracts from the Granadian PGI was demonstrated compared to conventional SLE. The proposed surface response model for PLE optimization showed the effect of the independent variables (temperature, time, percentage of ethanol, and S–S ratio) on the response variables (yield, total phenolic compounds, total phenolic acids, and total flavonoid content). Statistical analysis of the results confirmed that the proposed model was fitted, being appropriate for explaining the obtained results. Finally, the estimated optimum theoretical values of independent variables to maximize the response variables were calculated based on optimum extraction conditions. The chemical characterization of these extracts allowed the detection of 20 compounds, mainly belonging to phenolic acid and flavonoid families. The extraction yield found for the SLE was two times lower than the best PLE experimental condition, and a much lower value of phenolic compounds was reported for SLE than that obtained for all PLE extracts (up to 10 times lower in some comparisons). Coumaroylquinic acid was the only phenolic compound not recovered by conventional extraction.

It can be concluded that the optimized PLE for green asparagus is a powerful methodology for the recovery of the wide range of phytochemicals present in this Huétor Tájar PGI asparagus.

## Figures and Tables

**Table 1:** Tentatively identified compounds in the analyzed asparagus extracts.

**Peak**	RT ^1^ (min)	*m*/*z* (Exp)	*m*/*z* (Theor)	Error (ppm)	mSigma	Molecular Formula	Proposed Compound	PLE and SLE Extracts
**1**	3	191.0571	191.0561	−4.3	7.9	C_7_H_12_O_6_	Quinic acid	PLE *, SLE
**2**	9.2	353.0883	353.0878	−1.4	22.1	C_16_H_18_O_9_	Chlorogenic acid	PLE *, SLE
**3**	10.5	337.0915	337.0929	6.9	1.5	C_16_H_18_O_8_	Coumaroylquinic acid	PLE 1,2,3,4,5,6,7,8,9, 10,11,12,13,14,15,16,17, 18,19,21,22,23,24,25
**4**	11	367.1039	367.1035	1.1	21	C_17_H_20_O_9_	Feruloylquinic acid	PLE *, SLE
**5**	11.2	311.0007	311.0028	1.4	6.8	C_18_H_30_O_4_	UK 1	PLE *
**6**	11.5	355.101	355.1035	5.9	42	C_16_H_20_O_9_	Feruloyl hexose	PLE 1,2,3,4,5,6,7,8,9, 10,11,12,13,14,15,16,17, 18,19,23,24,25, SLE
**7**	11.8	311.0263	311.0256	−1.7	63.5	C_9_H_12_O_12_	UK 2	PLE *
**8**	12.3	311.0005	311.0031	−1.4	16.5	C_18_H_30_O_4_	UK 3	PLE *
**9**	12.6	771.2017	771.1989	−2.2	28.9	C_33_H_40_O_21_	Quercetin glucosyl rutinoside	PLE *, SLE
**10**	13.2	609.1562	609.1555	−0.2	25.6	C_27_C_31_O_16_	Rutin	PLE *, SLE
**11**	13.9	463.0883	463.0882	0.4	10.3	C_21_H_20_O_12_	Quercetin-glucoside	PLE *, SLE
**12**	14.1	593.1525	593.1512	−0.6	22.1	C_27_H_30_O_15_	Kaempferol-rutinoside	PLE *, SLE
**13**	14.2	623.1619	623.1618	0.7	19.4	C_28_H_32_O_16_	Isorhamnetin-rutinoside	PLE *, SLE
**14**	16	523.3837	523.2760	5.3	32.9	C_24_H_44_O_12_	UK 4	PLE *
**15**	21.1	329.2326	329.2333	3.7	12.5	C_18_H_34_O_5_	Trihydroxy-octadecaenoic acid	PLE *
**16**	21.3	383.1128	383.1136	4.2	1.1	C_21_H_20_O_7_	Dicoumaroylglycerol	PLE *, SLE
**17**	21.6	413.1239	413.1242	4.2	22.5	C_22_H_22_O_8_	Coumaroylferuloyl glycerol	PLE *, SLE
**18**	21.9	443.1325	443.1348	5.9	7.9	C_23_H_24_O_9_	Diferuloyl glycerol	PLE *, SLE
**19**	41.1	277.2161	277.2173	3.8	62.4	C_18_H_30_O_2_	Linolenic acid	PLE *
**20**	43.7	279.2536	279.2540	4	28.2	C_18_H_32_O_2_	Linoleic acid	PLE *

^1^ RT, retention time. UK, unknown compound. * All PLE extracts.

**Table 2:** Analysis of Variance (ANOVA) of the proposed regression models.

Y_1_					
*Variable*	*Sum of squares*	*d.f.*	*Mean Square*	*F-Ratio*	*p-Value*
X_1_: Temperature	1748.27	1	1748.27	3793.99	0.0103
X_2_: % EtOH	49.8912	1	49.8912	108.27	0.061
X_3_: Extraction time	265.038	1	265.038	575.17	0.0265
X_4_: S–S	20.6204	1	20.6204	44.75	0.0945
X_2_X_2_	140.293	1	140.293	304.45	0.0364
X_2_X_3_	46.6476	1	46.6476	101.23	0.0631
X_2_X_4_	487.792	1	487.792	1058.58	0.0196
X_3_X_3_	44.17	1	44.17	95.85	0.0648
X_3_X_4_	321.16	1	321.16	696.96	0.0241
X_4_X_4_	236.344	1	236.344	512.9	0.0281
Lack-of-fit	1416.09	13	108.93	236.39	0.0502
Pure error	0.4608	1	0.4608		
Total (corr.)	5048.77	24			
R^2^	0.719427				
**Y_2_**					
*Variable*	*Sum of squares*	*d.f.*	*Mean Square*	*F-Ratio*	*p-Value*
X_1_: Temperature	3725.07	1	3725.07	275.52	0.0383
X_2_: % EtOH	2980.15	1	2980.15	220.43	0.0428
X_3_: Extraction time	0.356199	1	0.356199	0.03	0.8976
X_4_: S–S	79.0719	1	79.0719	5.85	0.2496
X_1_X_1_	11.8109	1	11.8109	0.87	0.5215
X_1_X_2_	87.9975	1	87.9975	6.51	0.2378
X_1_X_3_	20.6351	1	20.6351	1.53	0.4332
X_1_X_4_	145.53	1	145.53	10.76	0.1883
X_2_X_2_	3.14735 × 10^−5^	1	3.14735 × 10^−5^	0	0.999
X_2_X_3_	120.993	1	120.993	8.95	0.2054
X_2_X_4_	851.39	1	851.39	62.97	0.0798
X_3_X_3_	19.0035	1	19.0035	1.41	0.4461
X_3_X_4_	1.05488	1	1.05488	0.08	0.8266
X_4_X_4_	11.0446	1	11.0446	0.82	0.5321
Lack-of-fit	750.45	9	83.3833	6.17	0.2992
Pure error	13.52	1	13.52		
Total (corr.)	9538.24	24			
R^2^	0.919905				
**Y_3_**					
*Variable*	*Sum of squares*	*d.f.*	*Mean Square*	*F-Ratio*	*p-Value*
X_1_: Temperature	2446.91	1	2446.91	221.54	0.0427
X_2_: % EtOH	2294.93	1	2294.93	207.78	0.0441
X_3_: Extraction time	1.01332	1	1.01332	0.09	0.8128
X_4_: S–S	40.7444	1	40.7444	3.69	0.3056
X_1_X_1_	1.21651	1	1.21651	0.11	0.796
X_1_X_2_	30.1739	1	30.1739	2.73	0.3464
X_1_X_3_	11.1687	1	11.1687	1.01	0.4982
X_1_X_4_	146.599	1	146.599	13.27	0.1705
X_2_X_2_	1.0669	1	1.0669	0.1	0.8082
X_2_X_3_	105.217	1	105.217	9.53	0.1995
X_2_X_4_	517.419	1	517.419	46.85	0.0924
X_3_X_3_	5.14163	1	5.14163	0.47	0.6188
X_3_X_4_	4.38077	1	4.38077	0.4	0.6422
X_4_X_4_	8.91063	1	8.91063	0.81	0.5341
Lack-of-fit	603.555	9	67.0617	6.07	0.3014
Pure error	11.045	1	11.045		
Total (corr.)	6793.25	24			
R^2^	0.909528				
**Y_4_**					
*Variable*	*Sum of squares*	*d.f.*	*Mean Square*	*F-Ratio*	*p-Value*
X_1_: Temperature	135.195	1	135.195	1081.56	0.0194
X_2_: % EtOH	44.5893	1	44.5893	356.71	0.0337
X_3_: Extraction time	2.4977	1	2.4977	19.98	0.1401
X_4_: S–S	6.54616	1	6.54616	52.37	0.0874
X_1_X_1_	5.70086	1	5.70086	45.61	0.0936
X_1_X_2_	14.8228	1	14.8228	118.58	0.0583
X_1_X_3_	1.53315	1	1.53315	12.27	0.1771
X_1_X_4_	0.00324709	1	0.00324709	0.03	0.8983
X_2_X_2_	1.11277	1	1.11277	8.9	0.2059
X_2_X_3_	0.49643	1	0.49643	3.97	0.2961
X_2_X_4_	41.479	1	41.479	331.83	0.0349
X_3_X_3_	4.74534	1	4.74534	37.96	0.1024
X_3_X_4_	1.15483	1	1.15483	9.24	0.2023
X_4_X_4_	0.125791	1	0.125791	1.01	0.499
Lack-of-fit	32.3611	9	3.59568	28.77	0.1418
Pure error	0.125	1	0.125		
Total (corr.)	299.83	24			
R^2^	0.891652				

**Table 3:** Comparison between experimental results and predicted values for PLE.

	Y_1_			Y_2_			Y_3_			Y_4_		
Run	Predicted	Exp.	CV	Predicted	Exp.	CV	Predicted	Exp.	CV	Predicted	Exp.	CV
PLE 1	17.80	20.03	8.3	64.8	68 ± 0.6	3.4	51.0	50.8 ± 0.7	0.3	13.8	17.2 ± 0.1	10.3
PLE 2	34.55	23.86	25.9	46.4	34 ± 1	17.3	35.5	26 ± 1	16.3	9.5	7.49 ± 0.06	12.5
PLE 3	26.08	15.6	35.6	70.2	69 ± 3	1.1	56.1	57 ± 3	1.7	12.3	11.6 ± 0.5	4.8
PLE 4	42.87	50.25	11.2	74.1	80 ± 5	5.6	59.6	63 ± 5	3.5	16.1	17.7 ± 0.7	5.7
PLE 5	40.51	43.8	5.5	42.5	54 ± 3	16.2	32.0	40 ± 2	15.2	12.3	13.9 ± 0.9	8.0
PLE 6	25.97	21.61	13.0	72.4	86 ± 2	11.8	57.5	67 ± 2	11.0	16.6	18.4 ± 0.3	5.1
PLE 7	39.05	46.29	12.0	44.2	59 ± 2	20.4	34.0	49 ± 2	17.0	11.7	9.6 ± 0.3	10.8
PLE 8	17.65	23.11	19.0	44.7	32.6 ± 0.1	17.0	33.4	24.5 ± 0.2	16.6	10.0	8.1 ± 0.2	11.4
PLE 9	24.62	23.64	2.9	71.9	52 ± 3	23.0	58.2	41 ± 3	19.5	11.8	10.8 ± 0.4	5.4
PLE 10	32.27	35.61	7.0	44.9	38.5 ± 0.7	10.8	34.9	29.3 ± 0.7	12.4	9.9	9.24 ± 0.05	3.5
PLE 11	26.54	29	6.3	43.7	41.2 ± 1.6	4.1	33.4	32 ± 2	2.7	10.3	9.0 ± 0.2	7.0
PLE 12	45.03	35.58	16.6	43.3	48 ± 1	7.0	33.9	38 ± 1	7.5	9.1	10.1 ± 0.4	6.1
PLE 13	37.99	37.52	0.9	46.2	48 ± 2	2.2	36.5	39 ± 2	4.2	9.5	9 ± 1	11.1
PLE 14	43.04	34.24	16.1	24.4	18.2 ± 0.6	15.8	17.0	11.8 ± 0.5	20.1	7.4	6.4 ± 0.1	7.9
PLE 15	32.27	36.57	8.8	44.9	43.7 ± 0.9	1.9	34.9	34.0 ± 0.7	1.9	9.9	9.7 ± 0.2	2.0
PLE 16	38.52	39.66	2.1	65.5	77 ± 9	11.3	52.8	64 ± 8	13.2	12.4	13 ± 1	8.6
PLE 17	41.61	43.29	2.8	46.6	42.5 ± 0.4	6.5	36.0	32.1 ± 0.7	8.1	10.7	10.4 ± 0.3	2.9
PLE 18	45.48	50.32	7.1	45.6	50 ± 2	6.9	35.8	41 ± 1	9.0	11.3	9.6 ± 0.2	8.6
PLE 19	54.06	70.01	18.2	19.6	22.4 ± 0.3	9.3	13.7	15.1 ± 0.5	6.8	4.2	7.3 ± 0.2	23.9
PLE 20	58.56	61.72	3.7	17.4	23 ± 1	13.4	12.3	15.1 ± 0.5	10.1	6.4	8 ± 1	18.4
PLE 21	60.02	58.34	2.0	15.7	12.9 ± 0.2	14.1	10.3	6.8 ± 0.2	22.3	7.0	6.0 ± 0.1	7.6
PLE 22	37.16	30.22	14.6	17.9	21 ± 1	11.8	11.7	15.1 ± 0.9	18.2	4.7	6.1 ± 0.1	11.7
PLE 23	62.38	50.93	14.3	47.3	45 ± 3	4.0	37.9	36 ± 2	2.9	10.8	8.3 ± 0.8	15.5
PLE 24	45.59	61.69	21.2	43.4	27 ± 3	28.0	34.4	20 ± 2	33.1	7.0	7.1 ± 0.2	1.8
PLE 25	46.73	33.38	23.6	25.1	31.9 ± 0.7	11.5	18.8	24.8 ± 0.8	13.0	6.0	7.15 ± 0.07	8.7

Exp. = Experimental. CV = coefficient of variation. Y_1_ = Extraction yield (%); Y_2_ = Total phenolic compounds (mg compound/g extract); Y_3_ = Phenolic acids (mg compound/g extract); Y_4_ = Flavonoids (mg compound/g extract).

**Table 4:** Theoretical values of independent variables to maximize the response variables provided by the model.

Factors	Temperature X_1_ (°C)	EtOH X_2_ (%)	Time X_3_ (min)	Sand—Sample Ratio X_4_ (*w*/*w*)	*Theoretical* *Optimum*
*Variable* *Response*					
Yield	184.1	94.5	34.7	5.7	89.5%
TPC	35.9	94.5	29.0	5.2	87.30 mg/g
Phenolic acids	35.9	94.1	31.5	5.7	71.02 mg/g
Flavonoids	36.1	94.5	5.8	5.7	21.00 mg/g
Multiple response	67.6	92.9	34.8	5.7	Yield = 66.4% TPC = 78.94 mg/g

TPC: total phenolic compounds, mg/g extract.

## Data Availability

Not applicable.

## Supplementary Materials

The following supporting information can be downloaded at www.mdpi.com/article/10.3390/metabo12100951/s1, Table S1: Experimental conditions of pressurized fluid extraction. Table S2: Calibration parameters. Table S3: Extraction yield of different PLE experiments (1–25) and SLE extraction. Table S4: Total and individual contents of phenolic compounds from the PLE and SLE extracts. Values are expressed as X ± SD in mg of compound/g extract.
