# Supplementary material for: Enhancing the Production of the Phenolic Extracts of Asparagus Using an Advanced Green Process
Source: Metabolites. 2022 Oct 6;12(10):951. doi: 10.3390/metabo12100951 (PMC9607466; doi:10.3390/metabo12100951)
Supplement: Supplementary file 1 [file metabolites-12-00951-s001.zip › metabolites-1861106-supplementary.pdf]

Table S1. Experimental conditions of pressurized liquid extraction.

Experiment	T[1] (°C)	Ethanol (%)	t[2] (min)	S-S (w/w)
PLE 1	36.00	50.00	20.00	3.50
PLE 2	60.00	20.00	30.00	5.00
PLE 3	60.00	80.00	10.00	2.00
PLE 4	60.00	80.00	30.00	5.00
PLE 5	60.00	20.00	10.00	2.00
PLE 6	60.00	80.00	10.00	5.00
PLE 7	60.00	20.00	30.00	2.00
PLE 8	60.00	20.00	10.00	5.00
PLE 9	60.00	80.00	30.00	2.00
PLE 10	110.00	50.00	20.00	3.50
PLE 11	110.00	50.00	5.00	3.50
PLE 12	110.00	50.00	20.00	1.00
PLE 13	110.00	50.00	35.00	3.50
PLE 14	110.00	6.00	20.00	3.50
PLE 15	110.00	50.00	20.00	3.50
PLE 16	110.00	95.00	20.00	3.50
PLE 17	110.00	50.00	20.00	6.00
PLE 18	160.00	80.00	10.00	5.00
PLE 19	160.00	20.00	30.00	5.00
PLE 20	160.00	20.00	30.00	2.00
PLE 21	160.00	20.00	10.00	2.00
PLE 22	160.00	20.00	10.00	5.00

PLE 23	160.00	80.00	30.00	5.00
PLE 24	160.00	80.00	10.00	2.00
PLE 25	184.00	50.00	20.00	3.50

[1] T: temperature.
[2] t: time.
[3] S-S: sand-sample ratio.

Table S2. Calibration parameters.

Standard	Calibration Range (mg/L)	Calibration Curve	R^2
Chlorogenic acid	0.10 - 10	y = 392180x - 86822	0,9897
Ferulic acid	0.10 - 75	y = 14525x + 230871	0,9937
Quercetin-glucoside	0.10 - 5	y = 1000000x + 41654	0,9934
Quinic acid	0.10 - 100	y = 361668x – 80932	0,9831
Rutin	0.10 - 100	y = 776936x + 43050	0,9958

Table S3. Extraction yield of different PLE experiments (1-25) and SLE extraction.

PLE Experiments	Yield (%)	PLE/SLE Experiments	Yield (%)
PLE 1	20.03	PLE 14	34.24
PLE 2	23.86	PLE 15	36.57
PLE 3	15.60	PLE 16	39.66
PLE 4	50.25	PLE 17	43.29
PLE 5	43.80	PLE 18	50.32

PLE 6	21.61	PLE 19	70.01
PLE 7	46.29	PLE 20	61.72
PLE 8	23.11	PLE 21	58.34
PLE 9	23.64	PLE 22	30.22
PLE 10	35.61	PLE 23	50.93
PLE 11	29.00	PLE 24	61.69
PLE 12	35.58	PLE 25	33.38
PLE 13	37.52	SLE	30.28

Table S4. Total and individual content of phenolic compounds in the PLE and SLE extract. Value expressed in mg compound/g dry extract as X ± SD

Compound	PLE 1	PLE 2	PLE 3	PLE 4	PLE 5	PLE 6	PLE 7	PLE 8	PLE 9	PLE 10	PLE 11	PLE 12	PLE 13
Chlorogenic acid	0.34 ± 0.02	0.55 ± 0.03	0.28 ± 0.02	2.42 ± 0.05	0.17 ± 0.01	0.55 ± 0.02	0.20 ± 0.02	0.605 ± 0.008	1.02 ± 0.07	0.74 ± 0.02	1.00 ± 0.05	1.12 ± 0.06	1.05 ± 0.06
Coumaroylquinic acid	0.59 ± 0.02	0.19 ± 0.02	0.279 ± 0.006	0.54 ± 0.04	0.289 ± 0.001	0.54 ± 0.03	0.266 ± 0.009	0.18 ± 0.02	0.379 ± 0.006	0.29 ± 0.02	0.34 ± 0.01	0.30 ± 0.02	0.20 ± 0.04
Feruloylquinic acid	24.6 ± 0.5	13 ± 2	23 ± 1	26 ± 2	17.0 ± 0.2	30 ± 2	17.1 ± 0.5	11.1 ± 0.3	16 ± 1	12.3 ± 0.6	13.8 ± 0.2	13.8 ± 0.6	14.0 ± 0.9
Feruloyl hexose	15.1 ± 0.3	4.6 ± 0.2	13.0 ± 0.7	17 ± 2	9.98 ± 0.02	16.1 ± 0.7	11.8 ± 0.7	3.6 ± 0.2	6.8 ± 0.3	6.3 ± 0.5	6.9 ± 0.1	6.3 ± 0.4	5.9 ± 0.3
Quercetin glucosyl rutinoside	4.04 ± 0.03	0.256 ± 0.003	0.491 ± 0.007	4.2 ± 0.1	3.66 ± 0.03	4.1 ± 0.1	0.402 ± 0.007	0.29 ± 0.01	0.48 ± 0.03	0.34 ± 0.01	0.42 ± 0.01	0.390 ± 0.008	0.33 ± 0.02
Rutin	12.6 ± 0.1	6.90 ± 0.06	10.6 ± 0.5	12.9 ± 0.5	9.8 ± 0.8	13.6 ± 0.3	8.8 ± 0.3	7.4 ± 0.2	9.8 ± 0.4	8.52 ± 0.04	8.2 ± 0.2	9.3 ± 0.4	8 ± 1
Quercetin-glucoside	0.092 ± 0.002	0.039 ± 0.006	0.061 ± 0.004	0.09 ± 0.01	0.050 ± 0.003	0.102 ± 0.002	0.050 ± 0.002	0.043 ± 0.002	0.086 ± 0.003	0.072 ± 0.003	0.070 ± 0.008	0.071 ± 0.005	0.070 ± 0.002

Kaempferol-rutinoside	0.197 ± 0.006	0.173 ± 0.007	0.193 ± 0.006	0.21 ± 0.02	0.15 ± 0.02	0.25 ± 0.01	0.146 ± 0.009	0.18 ± 0.01	0.21 ± 0.01	0.16 ± 0.01	0.171 ± 0.006	0.19 ± 0.01	0.184 ± 0.003
Isorhamnetin-rutinoside	0.252 ± 0.008	0.139 ± 0.004	0.21 ± 0.01	0.25 ± 0.01	0.181 ± 0.003	0.30 ± 0.01	0.160 ± 0.007	0.015 ± 0.002	0.24 ± 0.01	0.160 ± 0.03	0.183 ± 0.003	0.20 ± 0.02	0.157 ± 0.005
Dicoumaroylglycerol	0.51 ± 0.01	4.078 ± 0.008	9 ± 1	4.2 ± 0.2	0.63 ± 0.08	8.9 ± 0.3	8.9 ± 0.5	3.6 ± 0.3	5.0 ± 0.4	4.1 ± 0.3	3.7 ± 0.1	5.3 ± 0.3	5.7 ± 0.6
Coumaroylferuloyl glycerol	9.3 ± 0.3	4.4 ± 0.3	12 ± 2	12 ± 1	10.3 ± 0.2	11.5 ± 0.4	11 ± 1	4.9 ± 0.2	8.3 ± 0.9	5.5 ± 0.2	5.7 ± 0.4	6.9 ± 0.4	8.0 ± 0.9
Diferuloyl glycerol	0.37 ± 0.04	0.28 ± 0.02	0.50 ± 0.04	0.36 ± 0.05	0.343 ± 0.006	0.279 ± 0.004	0.280 ± 0.009	0.405 ± 0.004	3.6 ± 0.4	0.42 ± 0.04	0.43 ± 0.02	3.7 ± 0.4	3.61 ± 0.04
**Total Phenolic Acids**	50.8 ± 0.7	26 ± 1	57 ± 3	63 ± 5	40 ± 2	67 ± 2	49 ± 2	24.5 ± 0.2	41 ± 3	29.3 ± 0.7	32 ± 2	38 ± 1	39 ± 2
**Total Flavonoids**	17.2 ± 0.1	7.49 ± 0.06	11.6 ± 0.5	17.7 ± 0.7	13.9 ± 0.9	18.4 ± 0.3	9.6 ± 0.3	8.1 ± 0.2	10.8 ± 0.4	9.24 ± 0.05	9.0 ± 0.2	10.1 ± 0.4	9 ± 1
**Total Phenolic Compounds**	68.0 ± 0.6	34 ± 1	69 ± 3	80 ± 5	54 ± 3	86 ± 2	59 ± 2	32.6 ± 0.1	52 ± 3	38.5 ± 0.7	41.2 ± 1.6	48 ± 1	48 ± 2

Table S4 continuation. Total and individual content of phenolic compounds in the PLE and SLE extract. Value expressed in mg compound/g dry extract as X ± SD

Compound	PLE 14	PLE 15	PLE 16	PLE 17	PLE 18	PLE 19	PLE 20	PLE 21	PLE 22	PLE 23	PLE 24	PLE 25	SLE
Chlorogenic acid	0.4 ± 0.1	1.3 ± 0.3	1.5 ± 0.2	1.43 ± 0.07	0.94 ± 0.05	0.66 ± 0.04	0.70 ± 0.04	0.378 ± 0.002	0.41 ± 0.06	0.50 ± 0.05	0.67 ± 0.03	0.60 ± 0.03	0.209 ± 0.008
Coumaroylquinic acid	0.09 ± 0.01	0.27 ± 0.03	0.370 ± 0.008	0.25 ± 0.02	0.163 ± 0.004	0.11 ± 0.01	ND	0.059 ± 0.005	0.081 ± 0.002	0.15 ± 0.03	0.10 ± 0.02	0.13 ± 0.03	ND
Feruloylquinic acid	7.2 ± 0.4	14.6 ± 0.9	24.2 ± 0.4	15.7 ± 0.7	14.2 ± 0.4	8.4 ± 0.2	9.0 ± 0.6	5.3 ± 0.2	6 ± 1	11 ± 2	8.3 ± 0.9	9 ± 1	0.381 ± 0.006
Feruloyl hexose	0.18 ± 0.04	6.2 ± 0.5	7.9 ± 0.9	5.3 ± 0.7	5.2 ± 0.5	0.65 ± 0.06	ND	ND	ND	3.8 ± 0.4	0.39 ± 0.06	0.45 ± 0.04	0.089 ± 0.007

Quercetin glucosyl rutinoside	0.20 ± 0.01	0.373 ± 0.004	0.39 ± 0.02	0.407 ± 0.004	0.33 ± 0.01	0.25 ± 0.02	0.234 ± 0.008	0.161 ± 0.004	0.16 ± 0.02	0.122 ± 0.008	0.197 ± 0.009	0.209 ± 0.002	0.030 ± 0.002
Rutin	5.93 ± 0.08	8.9 ± 0.2	11 ± 1	9.5 ± 0.4	8.9 ± 0.2	6.7 ± 0.1	6.2 ± 0.3	5.7 ± 0.1	5.7 ± 0.1	7.8 ± 0.8	6.7 ± 0.1	6.7 ± 0.07	0.53 ± 0.06
Quercetin-glucoside	0.023 ± 0.003	0.064 ± 0.004	0.17 ± 0.01	0.073 ± 0.006	0.072 ± 0.002	0.050 ± 0.004	0.034 ± 0.003	0.040 ± 0.003	0.060 ± 0.001	0.09 ± 0.01	0.0819 ± 0.0005	0.082 ± 0.004	0.0060 ± 0.0004
Kaempferol-rutinoside	0.121 ± 0.001	0.196 ± 0.009	0.49 ± 0.04	0.23 ± 0.01	0.194 ± 0.007	0.130 ± 0.006	0.115 ± 0.006	0.084 ± 0.004	0.099 ± 0.002	0.173 ± 0.009	0.987 ± 0.008	0.110 ± 0.009	0.018 ± 0.001
Isorhamnetin-rutinoside	0.111 ± 0.005	0.177 ± 0.001	0.37 ± 0.02	0.224 ± 0.004	0.164 ± 0.007	0.12 ± 0.02	0.103 ± 0.005	0.074 ± 0.003	0.088 ± 0.001	0.13 ± 0.01	0.09 ± 0.01	0.090 ± 0.009	0.026 ± 0.001
Dicoumaroylglycerol	0.334 ± 0.006	4.7 ± 0.5	12 ± 2	3.6 ± 0.2	6.5 ± 0.4	0.50 ± 0.06	0.47 ± 0.04	0.32 ± 0.06	3.5 ± 0.3	6.4 ± 0.7	3.3 ± 0.4	4.3 ± 0.1	0.066 ± 0.006
Coumaroylferuloyl glycerol	3.3 ± 0.2	6.0 ± 0.2	15 ± 2	4.7 ± 0.2	9.3 ± 0.1	4.7 ± 0.4	4.4 ± 0.1	0.44 ± 0.08	5.3 ± 0.6	10.7 ± 0.9	6.8 ± 0.7	7.2 ± 0.3	0.097 ± 0.006
Diferuloyl glycerol	0.17 ± 0.01	0.54 ± 0.01	5.1 ± 0.5	0.34 ± 0.04	4.5 ± 0.4	0.4 ± 0.05	0.35 ± 0.02	0.28 ± 0.05	0.53 ± 0.03	4.6 ± 0.3	0.57 ± 0.04	3.4 ± 0.2	0.069 ± 0.004
**Total Phenolic Acids**	11.8 ± 0.5	34.0 ± 0.7	64 ± 8	3.1 ± 0.7	41 ± 1	15.1 ± 0.5	15.1 ± 0.5	6.8 ± 0.2	15.1 ± 0.9	36 ± 2	20 ± 2	24.8 ± 0.8	0.91 ± 0.02
**Total Flavonoids**	6.4 ± 0.1	9.7 ± 0.2	13 ± 1	10.4 ± 0.3	9.6 ± 0.2	7.3 ± 0.2	8 ± 1	6.0 ± 0.1	6.1 ± 0.1	8.3 ± 0.8	7.1 ± 0.2	7.15 ± 0.07	0.61 ± 0.06
**Total Phenolic Compounds**	18.2 ± 0.6	43.7 ± 0.9	77 ± 9	42.5 ± 0.4	50 ± 2	22.4 ± 0.3	23 ± 1	12.9 ± 0.2	21 ± 1	45 ± 3	27 ± 3	31.9 ± 0.7	1.52 ± 0.04
